# Bacterial Toxin-Antitoxin Systems’ Cross-Interactions—Implications for Practical Use in Medicine and Biotechnology

**DOI:** 10.3390/toxins15060380

**Published:** 2023-06-04

**Authors:** Lidia Boss, Barbara Kędzierska

**Affiliations:** Department of Bacterial Molecular Genetics, Faculty of Biology, University of Gdańsk, 80-309 Gdańsk, Poland; barbara.kedzierska@ug.edu.pl

**Keywords:** toxin-antitoxin, cross interactions, cross-talk, practical use, applications

## Abstract

Toxin-antitoxin (TA) systems are widely present in bacterial genomes. They consist of stable toxins and unstable antitoxins that are classified into distinct groups based on their structure and biological activity. TA systems are mostly related to mobile genetic elements and can be easily acquired through horizontal gene transfer. The ubiquity of different homologous and non-homologous TA systems within a single bacterial genome raises questions about their potential cross-interactions. Unspecific cross-talk between toxins and antitoxins of non-cognate modules may unbalance the ratio of the interacting partners and cause an increase in the free toxin level, which can be deleterious to the cell. Moreover, TA systems can be involved in broadly understood molecular networks as transcriptional regulators of other genes’ expression or modulators of cellular mRNA stability. In nature, multiple copies of highly similar or identical TA systems are rather infrequent and probably represent a transition stage during evolution to complete insulation or decay of one of them. Nevertheless, several types of cross-interactions have been described in the literature to date. This implies a question of the possibility and consequences of the TA system cross-interactions, especially in the context of the practical application of the TA-based biotechnological and medical strategies, in which such TAs will be used outside their natural context, will be artificially introduced and induced in the new hosts. Thus, in this review, we discuss the prospective challenges of system cross-talks in the safety and effectiveness of TA system usage.

## 1. Introduction

Toxin-antitoxin (TA) systems are intracellular modules consisting of a non-secreted stable toxin (most often a protein) and a susceptible to degradation antitoxin (either a protein or an RNA). According to currently proposed classification, TA modules are divided into eight types depending on the antitoxin chemical nature and its mode of action (listed in Table 1). Representative examples of TA systems belonging to each type are thoroughly reviewed in [1,2].

Although TA modules are abundant in bacterial and archaeal genomes, they are highly heterogeneously distributed among species and strains of microorganisms. Diverse and constantly improved bioinformatic approaches, as well as rapidly expanding bacterial genome databases, lead to discovery of numerous novel TA systems. A recently developed TASmania database enabled in silico identification of over two million candidates of TA loci, including orphan toxins and antitoxins [3]. TA systems had most likely undergone an exchange during evolution events as the prevalence of highly similar TA xenologs in bacterial chromosomes, prophages, and plasmids is observed. The chromosome-encoded TA cassettes are usually flanked by genes or pseudogenes related to diverse mobile genetic elements (MGE), such as genomic islands (GIs), transposons, integrative and conjugative elements (ICEs), as well as cryptic and active prophages which all constitute bacterial mobilome [4,5,6]. Thus, it was proposed that the TA systems are mostly related to MGE and can be acquired through horizontal gene transfer (HGT) [7]. The high prevalence of TA systems often leads to a situation where multiple TA modules, including homologous TA systems, coexist within a single microbial cell or even a single DNA particle. This may result in the TA modules’ cross-talk, understood either as the toxin neutralization by a non-cognate antitoxin (I), modulation of the toxin and/or antitoxin expression by components of a non-cognate TA system (II), or as a synergetic activity of multiple toxins resulting from a cascade of TA systems’ activation (III). Interestingly, despite the fact that multiple TA systems are frequently found within bacterial genomes, the episodes of their cross-talk are relatively rarely encountered [7,8]. Nonetheless, it seems highly possible that the events of non-cognate TA systems component cross-interaction can affect the evolution of those small genetic modules [7,9,10].

The toxin-antitoxin systems can be easily acquired through horizontal gene transfer, and at least some of them can possibly cross-regulate. Therefore, there is an apparent possibility that the TA modules’ activity can be disturbed by several factors in microorganisms living in their natural environment. For example, that activity can be affected through interference with the TA modules of other bacteria persisting within the environment or with the antitoxins encoded within phages’ genetic material. Moreover, TA systems can be involved in broadly understood molecular networks: for instance, as transcriptional regulators of other genes, when antitoxin or TA complex binds the promoter region and affects transcription of a given gene [11,12,13], or as factors directly affecting other genes’ expression through the toxin activity (e.g., due to mRNA degradation by the RNase toxin) [14,15]. This implies a question on the possibility and consequences of the TA system cross-interactions, especially in the context of the practical application of TA-based biotechnological and medical strategies.

Furthermore, multiple TA systems encoded within bacterial chromosomes could be considered a significant part of the genetic pool to form new components essential for bacterial adaptation in new ecological niches. Considering the fact that the selective pressure may provoke TA system differentiation, as well as their convergent evolution leading to cross-talk between non-cognate TA systems, the security of TA-dependent biosafety control methods should be especially taken into consideration. Therefore, the evolutionary potential of TA cassettes should also be considered when planning TA-based biotechnological and medical applications, as well as during the development of biosafety and crop preservation strategies.

To conclude, we are convinced that the TA modules might be considered as both a substantial gene source for the development of beneficial innovative biotechnological tools and approaches and as unpredictable components of those. Thus, in this review, we intend to highlight the subject of possible TA systems’ cross-talk and discuss the implications of the putative effects of such cross-interactions in the practical use of TA modules.

In the next sections, we are giving a short overview of the proposed mechanisms of TA systems’ activation and their possible biological function and consider the modes of TA systems’ cross-talk. This is followed by a description of proposed plans for TA systems’ practical use in medicine and biotechnology, presented in the context of the non-cognate modules’ possible cross-interactions. Finally, we discuss the potential role of other factors that could affect the activity of TA systems in TA-based biotechnological approaches.

**Table 1:** Classification of the toxin-antitoxin systems.

TA System Type	Antitoxin/Toxin Chemical Nature	Antitoxin Mode of Action
I	sRNA/Protein	Silencing toxin expression [16]
II	Protein/Protein	Formation of neutralizing complex due to direct binding to toxin [17]
III	Small pseudoknot RNA/Protein	Formation of neutralizing complex due to direct binding to toxin [18]
IV	Protein/Protein	Direct interaction with the toxin’s target and its protection [19]
V	Protein/Protein	Degradation of toxin mRNA [20]
VI	Protein/Protein	Direct binding to toxin and targeting it to proteolysis by ATP-dependent proteases [21]
VII	Protein/Protein	Toxin inactivation due to its post-translational modification [22]
VIII	sRNA/sRNA	Silencing toxin expression [23]

## 2. An Overview of Activation and Biological Function of the Bacterial Toxin-Antitoxin Systems

### 2.1. Toxin-Antitoxin System Classification and Distribution Issues

Since TA discovery, there have been many attempts to classify these systems; however, an ever-increasing number of new records made each classification method imperfect and unsatisfying due to the great structural and functional diversity of toxin and antitoxin components. Based on the nature and mode of action of the antitoxin, TA systems are currently classified into eight types, numbered according to their discovery (Table 1). Type II is the most diverse and numerous, while the other TA types are much less heterogeneous, with types V and VI being represented by only one example so far. However, even this classification system is not perfect, as some instances of cross-type features are observed. An example is *darTG*, a type II/IV TA hybrid system in which the DarG antitoxin inhibits expression of the DarT toxin via direct interaction, like in type II TAS, but also acts on the target of DarT removing toxic modification from the DNA, like in type IV TAS [24,25]. Moreover, some kind of shuffling between different types of TA systems, in terms of the structure and functionality of their components, has also been observed. Namely, ToxN toxin from *toxIN* type III TA structurally belongs to the CcdB/MazF super-family of type II toxins, while SymE type I toxin possesses structural similarities to MazE/AbrB super-family of type II antitoxins, indicating that a toxin may evolve from an antitoxin [26,27]. Furthermore, the YeeU antitoxin of the type IV YeeUV TA system is structurally similar to the ribonucleolytic type II toxins YoeB and RelE, showing another example that toxins and antitoxins can evolve from each other. As a proof of concept, an artificial toxin has been created from the native type V GhoS antitoxin with only two amino acid changes, and subsequently, two disparate antitoxins derived from type III ToxI and type II MqsA have been made to neutralize its toxicity [28].

Based on primary sequence and three-dimensional structure similarity, type II toxins and antitoxins have been grouped into families and super-families, which are not related, while sharing some functional connections. Several bioinformatic analyses revealed that the binding of toxins to specific antitoxins is often evolutionarily unpredictable and reflects the swapping of functional domains between different TA families or even between TA counterparts. Such is the case of the Phd antitoxin, which exhibits the same structural fold as the YefM antitoxin; however, both of them inhibit structurally unrelated toxins, i.e., Doc and YoeB, respectively. Furthermore, two pairs of toxins (MazF/CcdB and RelB/ParD) have a similar three-dimensional structure, although they exhibit different activities, i.e., RNA interferase in the case of MazF and RelB and gyrase inhibitor for CcdB and ParD.

All of the above examples highlight the great plasticity of TA components and support the idea that antitoxins and toxins were assembled on the basis of the “mix and match” rule, an event that happened multiple times in the course of evolution [29,30]. There are also at least two examples linking TA modules and bacterial secretion systems, suggesting that toxins could be converted into secreted effectors. Namely, *Bartonella* T4SS and *Xanthomonas* T3SS protein effectors derive from FicT and ζ toxins, respectively [31,32]

The number of identified TA loci varies greatly among different bacterial species. The data acquired from TAfinder, which is a type II TA prediction tool integrated into Toxin-Antitoxin DataBase (TADB 2.0), show that 66% of the analyzed species harbor at least one to up to twenty loci in a single strain, while 20% of species carry even more than twenty toxin-antitoxin modules. According to this database, the current record is held by *Microcystis aeruginosa* NIES-843 cyanobacterium, with its 113 predicted TA elements, followed by the human pathogen *Mycobacterium tuberculosis* H37Rv, in which 76 TA elements have been predicted [33]. Since TADB 2.0 database contains only records that match type II TA families that have been previously experimentally characterized, it could be expected that the total number of chromosomal TA elements in prokaryotic genomes is much larger. Accordingly, the TASmania database predicts as many as 423 and 269 putative TA components in the genomes of *M. aeruginosa* NIES-843 and *M. tuberculosis* H37Rv, respectively [3]. This shows that our knowledge of TA systems can be compared to the tip of the iceberg, where the majority of data is yet to be uncovered. Moreover, different analyses indicate that the TA systems are inconsistently and unevenly distributed across prokaryotic genomes and generally are not conserved in closely related species or even strains [4,34,35]. Analyses of the flanking genes and broader genetic context of different *E. coli* genomes suggest that their TA modules have been independently integrated and propagated within species through horizontal gene transfer mechanisms and homologous or non-homologous recombination events, which may be a general rule in the distribution of TA cassettes [35].

### 2.2. Mechanisms of TA Systems’ Activation

Expression of the toxin and antitoxin encoding genes has to be balanced to ensure that the toxic effect of each toxin molecule is neutralized by its associated antitoxin. Disruption of the toxin and antitoxin intracellular equilibrium results in the TA system activation. Until recently, the TA system activation was equated with transcriptional induction of the TA operon, especially since it was observed that many TA modules are transcriptionally activated during diverse cellular stresses [36,37,38,39]. However, the latest data indicated that although stress can induce TA transcription, it is not necessarily accompanied by toxin activity [40,41]. Therefore, in this review, we define the TA system activation as a situation where the toxin is active and affects its cellular target, leading to bacterial growth inhibition or even cell death.

The exact **mechanism of toxin activation** remains ambiguous. In most cases, it is probably achieved by enrichment in the intracellular amount of free toxin molecules. This could be accomplished either as a result of changes in the TA system gene expression, protease or RNase-mediated antitoxin degradation (for example, after the TA-encoding plasmid loss), via TA complex disruption (e.g., caused by binding of a third factor, such as a phage protein), or a compilation of these factors. In fact, it was proposed that in the case of the type III *toxIN* system, activation can be induced by bacterial transcription shutdown during T4 phage infection, resulting in unstable antitoxin (sRNA) depletion. The active ToxN protein would then block bacteriophage replication via cleavage of the phage’s RNA and the remaining bacterial transcripts, consequently leading to an abortive phage infection [42,43]. Similarly, a transcription-related mechanism of toxin activation was observed in the case of the type VII tripartite *pfkA/B-pfpC* system during biofilm formation by *Pseudomonas aeruginosa* PAO1 [44].

Likewise, a transcription-based mechanism of toxin activation can be true for certain type I TA systems. Namely, this type of activation was suggested for the type I *hokW-sokW* module of the Sakai prophage 5 (Sp5) encoded within the genome of *E. coli* O157:H7 Sakai strain. It was demonstrated that mitomycin C (MMC) treatment of bacteria resulted in Sp5 induction, combined with a diminution of *sokW* RNA and an increase in HokW toxin level. The MMC damages DNA and promotes RecA protein activation, which was shown to be engaged in DNA repair processes and lambdoid prophage induction. It was proposed that the depletion of *sokW* was mainly caused by transcription inhibition. Interestingly, an increase in HokW level was the combined effect of *sokW* depletion and some indefinite RecA-dependent processes. The HokW protein bound to the cell membrane promoted its depolarization and, as a result, cell growth retardation. It was observed that bacterial growth retardation after Sp5 induction was significantly lower in an *E. coli* strain with the *hokW-sokW* operon deletion, which suggests an effective HokW activation during Sp5 prophage lytic development [45].

On the other hand, examples of TA system activation that is probably mainly based on antitoxin degradation were described as well. Recently published data suggest that in the case of type II systems, *parDE*, and *mqsRA*, the toxin can be activated by ClpAP and ClpXP-mediated antitoxin degradation, respectively. In both cases, the process of antitoxin recognition and targeting to proteolysis was regulated by an additional factor [46,47].

Nevertheless, there are several TA examples, including a fused type II TA module (*capRel^JS46^*) and a two-partite *mazEF* module, where it is the direct binding of different phage capsid proteins to one of the TA system components that trigger the toxin activation [48,49,50,51]. Furthermore, Zhang et al. proposed lately that the protease-mediated toxin activation could also be the result of the protease’s direct binding to the TA complex, followed by the toxin release—even without the actual antitoxin proteolysis [48].

To conclude, it appears that there are several possible molecular pathways of toxin activation depending on the TA type and/or its biological role and that the process may be highly complicated and dependent on multiple cellular and environmental factors.

### 2.3. Contradictions Regarding Biological Function of TA Systems

Regardless of the concerns about the mechanism of TA systems’ activation, these modules were claimed to be engaged in a number of different processes and phenomena, such as genomic stabilization (including plasmid propagation and prophage lysogeny control), abortive phage infection, stress response modulation, virulence, and biofilm formation. However, lately, the most popular opinion is that despite the prevalence of TA systems, the majority of TA modules have little or no biological role. It is highly possible that at least some of the TA systems act as selfish entities, ensuring their own transmission, even if this has either no or a negative effect on the host fitness [52,53,54]. Since a lot of contradictions on this subject have accumulated over the years of research, currently, the actual **biological role of TA modules** is under intense debate [55,56,57,58,59,60].

For example, it has been shown that contrary to former reports [61,62,63], TA modules are not required for persister cell formation, neither in *S. enterica* nor in *E. coli* [64,65,66]. Likewise, deletion of the chromosomally encoded TA systems in *E. coli* does not seem to confer any selective advantage or disadvantage under stress conditions [67]. Moreover, there is a body of evidence that transcriptional activation of TA-encoding genes observed during stress is not necessarily accompanied by toxin activity [41].

On the other hand, several studies have shown convincing examples favoring the hypothesis of the TA system activity as a bacterial physiology modulator [39,62,68,69,70]. Thus, although a growing body of data suggest that TA modules are rather selfish genes, their role as the stress response or virulence modulators during microorganism-host interactions cannot be entirely excluded.

It is believed that in the case of plasmids, the TA systems have a clear role in their vertical inheritance by selectively inhibiting or slowing down the growth of plasmid-free daughter cells due to the toxin’s activity (the so-called **addiction phenomenon**) [71]. The first such module, named *ccdAB*, was discovered in the F plasmid of *E. coli* and was later shown to mediate the genetic stabilization of this mobile element by the PSK mechanism (Postsegregational Killing) [72,73]. According to this mechanism, degradation of the labile antitoxin and the lack of its *de novo* synthesis leads to the release of the stable toxin in the daughter cells devoid of a plasmid. The toxin then interacts with its intracellular target, leading to cell death or inhibition of metabolic processes. Thus, as progeny is eliminated from the population when the plasmid is lost, bacteria become “addicted” to TA modules located on plasmids.

Similarly, multiple examples of TA-mediated stabilization of other types of mobile genetic elements (MGE) have been shown [74,75,76,77,78]. Interestingly, the TA systems neither affect the likelihood of plasmid inheritance nor the number of plasmid-containing cells in a population. The successful TA-mediated MGE stabilization seems to be the direct effect of a toxin-dependent exclusion of daughter cells that did not inherit the TA module. Thus, the MGE stabilization effect of TA system activity is mainly considered a mechanism affecting competition between TA^+^ and TA^−^ mobile genetic elements and is one of the processes that drive the evolution of MGE and TA modules per se. Consistent with the model described above (first proposed by Cooper and Heinemann), the exclusion phenomena disappeared when the host cells were made immune to the effect of TA-mediated plasmid loss [79]. This kind of “immunization” results from the presence of other TA modules encoded within the host genome. For example, antitoxins of the chromosomal type II TA systems, *ccdAB_Ech_* and *ataRT*, were shown to, respectively, counteract the CcdB_F_ and AtaT_Pl_ toxins encoded within an invading plasmid DNA. It was shown that the cross-interaction between these chromosome and plasmid-born proteins prevented stabilization of the plasmid within bacterial cell populations (the so-called **anti-addiction phenomenon**) [9,80].

The anti-addiction effect of TA systems seems to imply their direct role in the protection of the bacterial cell from the invading MGEs that encode homologous TA systems. However, the abundance of TA systems in bacterial and archaeal genomes, and the commonly encountered co-existence of multiple homologous TA cassettes within a single DNA particle, combined with relatively rarely observed anti-addiction episodes, suggests that the effectiveness of TA systems as anti-addiction modules is rather modest. Therefore, such a phenomenon should be considered a unique example of an ongoing evolutionary process driven, among other factors, by the non-cognate toxin and antitoxin interactions. Following this path, an interaction between components of the non-cognate TA systems would result in the systems’ divergence or, at least in some cases, the elimination of one of the interacting modules [2,7].

This hypothesis could explain several documented examples of TA systems’ degeneration [4,10,81]. Moreover, it remains in line with the presumptive pattern of toxins’ and antitoxins’ evolution, recently proposed on the basis of data obtained from type II TA proteins’ co-evolution experiments. Namely, it was suggested that the common path of TA systems’ evolution includes changes in one protein that first broaden its specificity, allowing its partner to adapt, followed by the emergence of a specificity-restricting mutation [82]. Therefore, it seems that although the TA systems’ coevolution probably leads to the divergence of TA modules, it happens through the stage of TA components’ versatility [9,82,83]. Considering this point of view, it seems possible that interactions between non-cognate TA systems encoded within a single bacterial genome are far more prevalent than those observed experimentally. The main cause of this untraceability is probably due to the selective pressure, which provokes a rapid differentiation of the interacting modules.

Another example of the TA systems’ recognized biological function is their role as **phage-defense modules**. The subject of TA cassettes’ activity as the phage-defense modules and the phage counter-defense systems was thoroughly reviewed recently [84]; below, we are giving just a general description. Inhibition of phage propagation is well documented for TA cassettes of different types. For example, is was shown that the R1 plasmid-encoded type I *hok-sok* module has a role in T4 phage exclusion. This module is probably activated due to an sRNA antitoxin expression inhibition combined with its degradation after host transcription shut off [85]. The Hoc protein then binds to the cell membrane leading to its depolarization and premature cell death; thus, this reduces the amount of phage progeny and limits phage propagation. A similar mechanism of activation was proposed for the type III *toxIN_Pa_* module from *P. atrosepticum* in the course of ϕM1 infection [86]. Likewise, the *E. coli* gene expression switch off by the T4*∆dmd* phage leads to abortive infection mediated by the toxins of type II chromosomal *rnlAB* and plasmid-encoded *lsoAB* systems. It was observed that both systems are activated during the late stage of infection in a protease (ClpXP and/or Lon) dependent manner [87,88]. Interestingly, phage inhibition can be probably caused not only by the toxin activity but also due to the inhibition of phage gene transcription mediated by binding of the antitoxin to the phage DNA, as was proposed for the type II *crlAT* system of *P. aeruginosa* WK172 strain [89].

The anti-bacteriophage activity of TA modules may also be associated with **competition between bacteriophages**. Activation of the fused type II *capRel^SJ46^* system, encoded within the temperate prophage SJ46 genome, results in the lytic SECϕ27 phage abortive infection. CapRel^SJ46^ is activated by direct binding of the SECϕ27 phage Gp57 capsid protein, which probably induces conformational changes that, in turn, activate toxicity. The active CapRel^SJ46^ protein phosphorylates tRNAs and thus inhibits both the host and phage translation [48].

In contrast, some bacteriophages encode **pseudo-antitoxins** that can act to prevent the above-described TA-based anti-phage strategy [88,90,91]. For example, it was shown that the wild-type T4 bacteriophage encodes a *dmd* antitoxin which directly interacts with the RnlA and LsoA toxins, neutralizing their toxic effect [88]. Moreover, T4 encodes the PinA protein that directly binds and inhibits the Lon protease activity, which may affect the TA modules’ activation process [88,92]. Almost exactly the same mechanism of action was proposed for the 4.5 gene product of T7 phage that prevents type II system *sanaTA* -mediated abortive infection [93], for the *pseudo-toxI* sRNA encoded within phage ϕTE of *P. atrosepticum* [90] and for the anti-DarT1 factor encoded within some T-even phages [91].

It was also proposed that the bacterium-phage interaction constitutes a strong selective pressure for bacteriophages susceptible to TA-mediated exclusion to acquire a cross-interacting pseudo-antitoxin. On the other hand, an opposite trend was predicted for the host bacteria. Namely, it was suggested that the rise of a TA-resistant escape phage would drive the selection of non-cross-interacting toxins in bacteria [84]. All in all, it seems clear that the biological composition of the bacterial environment is the main factor affecting the TA modules’ activity and evolution.

## 3. Modes of the Toxin-Antitoxin System Cross-Talk

### 3.1. Specificity Determinants Allow Desired Interactions between the Toxin and Antitoxin

The ubiquity of different homologous and non-homologous TA systems within a single bacterial genome raises questions about their potential cross-interactions. Phylogenetic studies show that proteins of distantly related TA systems usually do not exhibit any cross-talk, whereas proteins of close homologs are sometimes able to interact with non-partners [94]. However, the latter situation presumably exemplifies an intermediate step in the evolution pathway toward complete divergence and insulation of paralogous systems in the same genome. This is confirmed by the observation that identical TA cassettes are mutually exclusive and virtually do not coexist in the same cell [35].

Given the danger brought by toxins to the bacterial cell and the fact that numerous different toxin-antitoxin cassettes can co-occur in a single genome on chromosomes, plasmids, or bacteriophages, it was crucial for TA systems to develop mechanisms that allow them to avoid non-specific, random cross-reactions. On the other hand, it was also critical for paralogous TA pairs to proceed to a safe evolution of insulation which is inevitably directed by genetic drift and selection based on the toxin effect. Thus, the biggest challenge for the interacting proteins is to keep the desired interaction and, at the same time, to avoid unwanted cross-talk, which can be dangerous to a cell. This balance is achieved by a subset of specificity-determining residues, which are responsible for the stabilization of cognate interactions and the disturbance of non-cognate ones [95,96].

The exact number of amino acid residues controlling the specificity of toxin-antitoxin pairing in the studied examples varies. In the case of two insulated *parDE* systems, three amino acid substitutions were shown to confer the toxin-antitoxin interaction specificity. Each of these key interface positions was shown to form a positive element that promotes the cognate interaction, while two of them serve as negative elements that protect from the non-cognate interaction [82]. However, substituting just one of these residues was sufficient to produce promiscuity [95]. Similarly, a single Asp83Tyr substitution in the Txe toxin of the type II *axe-txe* system was shown to relax the specificity enough to allow its interaction with the YefM antitoxin of the homologous *yefM-yoeB* TA system [97].

As an example of the hyper versatility of antitoxins, Kurata and co-authors described an antitoxin protein domain, DUF4065, that is able to form complexes with dozens of different cognate toxins. Moreover, through directed evolution, they were able to select variants that neutralize even a noncognate and non-homologous toxin with just two amino acid substitutions. Such antitoxins were called PanA, while this hyper-promiscuous domain has been named Panacea to underline its universality [98]. On the other hand, in their recent paper, Grabe and co-authors found that insulation of three *tacAT* paralogs from *Salmonella* spp. is conferred by the presence and size of a specific helix within the toxin structure. They also revealed that TacA antitoxins display remarkable flexibility in their structural organization, allowing the formation of additional interfaces within the toxin to accommodate this helical element. These additional helices on the TA interfaces may provide a safe space for the evolution of insulation between paralogous *tacAT* systems [99].

To summarize, the presence of promiscuous variants enabled by the superplasticity of the antitoxins seems to promote diversification which is crucial for the safe expansion of TA systems during evolution. Moreover, additional TA interfaces between both interacting partners act as elements supporting neutralization and compensate for imperfect fits between the highly evolving regions of the toxin and antitoxin.

### 3.2. Different Levels of Cross-Interactions between TA Partners

**Figure 1:**
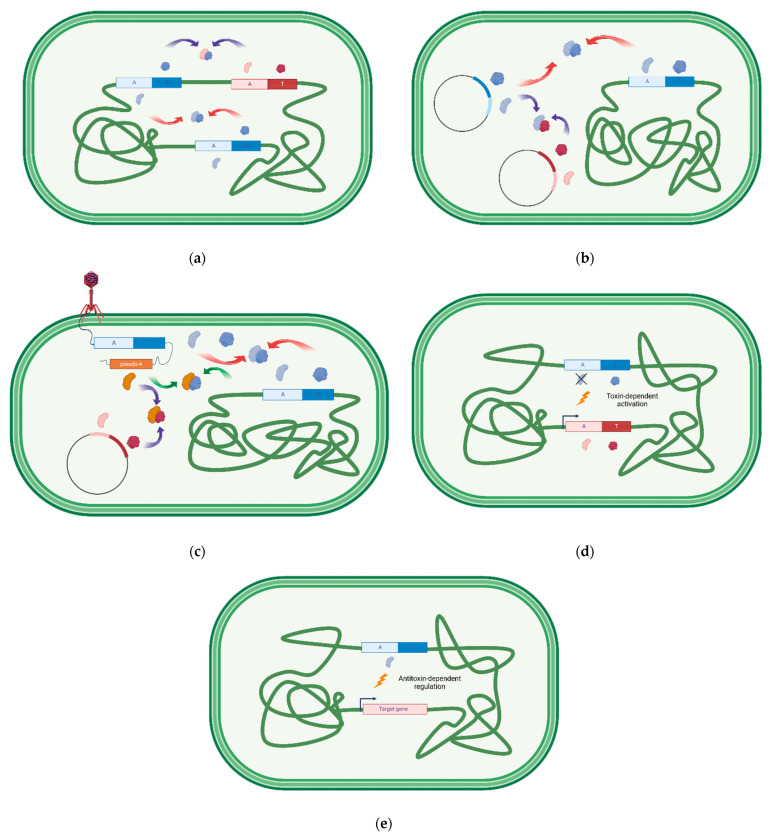
Possible modes of cross-talk between proteins belonging to different type II toxin-antitoxin systems. Elements of homologous and non-homologous TA systems are marked in blue and red, and pseudo-antitoxins are marked in orange. (**a**) Direct cross-interaction between proteins of homologous and non-homologous TA systems encoded within bacterial chromosomal DNA. (**b**) Direct cross-interaction between proteins of homologous and non-homologous TA systems encoded within plasmid and chromosomal DNA. (**c**) Direct cross-interaction between proteins of TA systems encoded within bacterial and phage genomes. (**d**) Indirect TA systems’ cross-activation. (**e**) TA-dependent regulation of an unrelated gene. Created with BioRender.com (https://app.biorender.com accessed on 2 June 2023).

Unspecific cross-talk between toxins and antitoxins of non-cognate modules may unbalance the ratio of the interacting partners and cause an increase in the free toxin level, which can be deleterious to the cell. Thus, multiple copies of highly similar or identical TA systems are rather infrequent and probably represent the transition stage in the course of evolution to the complete insulation or decay of one of them. Nevertheless, several types of cross-interactions have been described in the literature to date (Figure 1)—between homologous or non-homologous toxins and antitoxins from the same TA type, as well as between toxins or antitoxins belonging to different TA types. Another differentiation in the cross-talk types is based on the location of the TA systems—whether they are on the same or different replicons, for example, the chromosome, plasmid, or phage DNA. However, cross-interactions can occur not only directly between the toxin and the antitoxin but also at the regulatory level, where antitoxin alone or in the complex with its cognate toxin regulates the expression of another TA operon. Finally, TA interactions with other cellular proteins or the influence of other proteins on TA regulation have to be taken into account as well. We collected some examples of TA interactions and classified them according to their original location, as well as to the type of cross-talk they exhibit (Table 2, Figure 1).

#### 3.2.1. Examples of Cross-Talk between Proteins of Chromosomal TA Cassettes

A few examples of cross-talk between chromosomal TA cassettes were described for the *M. tuberculosis* H37Rv strain, which possesses 76 identified type II systems, including at least 50 *vapBC*, 10 of *mazEF*, and 3 of *relBE* families, respectively [119]. It was shown that within its three *relBE*-like modules, *relBE*, *relFG*, and *relJK*, that RelB and RelF antitoxins can directly interact with each of the three RelE-like toxins [101]. Moreover, it was demonstrated that the MazE1 antitoxin could reverse the toxicity of non-cognate MazF3 and MazF9 toxins, while the toxicity of MazE3 could be additionally inhibited by non-homologous VapB24 or VapB25 antidotes [100,102]. Different examples of non-cognate homologous and non-homologous interactions were also described for chromosomal TA modules of *Bifidobacterium longum*, in which MazF1 toxin was shown to directly interact with MazE2 and RelB antitoxins [103]. Similarly, RelB2 can act as an antidote for non-cognate RelE1 toxin in the *Yersinia pestis* cells [104].

#### 3.2.2. Cross-Interactions between TA Proteins Located on Different Replicons

Another level of cross-talk can occur between TA systems located in different replicons. There are several examples of cross-interactions between homologous chromosomal and plasmid TA components. Two instances apply to the *ccdAB* module. The chromosomally encoded *ccdAB* system of *Erwinia chrysanthemi* protects the cell from toxicity mediated by the F1 plasmid *ccdB* module [9]. Similarly, the CcdA antitoxin of F-related virulence plasmid (pO157) is able to counteract the chromosomal CcdB toxin of the *ccdAB* TA module found in some *E. coli* O157:H7 isolates [120]. Additionally, in some isolates of *E. coli*, plasmid-encoded AtaT toxin of the *ataRT* toxin-antitoxin cassette was inhibited by chromosomal AtaR antitoxin [80]. Moreover, Gog and co-workers observed cross-interactions between the KacA3 antidote located on a plasmid and chromosomal KacT2 toxin in *Klebsiella pneumoniae* [121]. Weak interactions were also identified between the Kid toxin of the *kis-kid* locus derived from the R1 plasmid and its chromosomal homolog—the *E. coli* MazE antitoxin [105].

There are also known examples of cross-talk between chromosomal and phage-encoded TA systems. The proteins of the chromosomal *phd-doc* cassette identified in *V. cholerae* were shown to cross-talk with the canonical P1 phage Phd-Doc complex [107]. A specific type of such interaction is exemplified by the *E. coli rnlAB* system, which makes these bacteria partially resistant to P1 phage infection. On the other hand, the T4 phage encodes the Dmd protein, which acts as a heterologous antitoxin of RnlA toxin and thus enables successful viral propagation [108]. A similar mechanism has been described for enterohaemorrhagic *E. coli* O157:H7 harboring pOSAK1 plasmid. This plasmid encodes the *lsoAB* TA module that is homologous to the K-12 *rnlAB* cassette. In this case, the Dmd protein can also directly suppress the toxin (LsoA), making these bacteria susceptible to T4 phage infection [88]. Finally, there is an example of cross-talk between two TA systems derived from different plasmids. Namely, the Kis antitoxin of the R1 plasmid was shown to inhibit toxicity mediated by CcdB encoded by the F plasmid, although with low efficiency [106].

#### 3.2.3. Antitoxins as Regulators of Unrelated Gene Expression

Typically, type II antitoxins alone or in complex with their cognate toxins act as repressors of their own promoters. They recognize specific DNA motifs within the promoter region and bind to it through their N-terminal domain. Interestingly, some examples of cross-regulation between two different TA systems have been characterized. It was shown that the CopA antitoxin of *parE/copA* system from *Shewanella oneidensis*, apart from its own autoregulation, also represses transcription from the *pemK/pemI* module found in the pMR-1 megaplasmid [76]. Another known case is represented by two homologous TA systems—*yefM-yoeB* and *axe-txe*, derived from *E. coli* chromosome and enterococcal pRUM plasmid, respectively. It was shown that the proteins of both cassettes are able to bind to their cognate and non-cognate operator regions with similar efficiency, although different repression level was observed for each promoter. That was due to the diverse location of both operator sequences with respect to the promoter sequences, and thus repression occurred at different steps of the transcription initiation process [113]. In both mentioned examples, the DNA motives that are recognized by antitoxins are highly similar within a pair of the two interacting modules.

Another example of interaction between TA systems is cross-regulation, that involves two TA modules belonging to different types. In the genome of the clinical isolate of *Enterococcus faecalis*, a locus encoding two adjacent, divergently organized TA modules was discovered. One of these modules is related to the type I *txpA-ratA* family, while the other belongs to the type II *mazEF* family [122]. The MazEF complex was shown to repress its own promoter and to activate the expression of RatA antitoxin using a common sequence present within both promoters [122].

Additionally, some antitoxins have been shown to have the capacity to regulate the expression of specific genes which are unrelated to TA systems in addition to their own operons [123]. For example, a MqsRA-like palindrome was found upstream of the transcription initiation site within the promoter of the *csgD* gene. The MqsA antitoxin was shown to directly bind and thereby repress expression of the *csgD* gene that is a master regulator of biofilm formation, which controls curli and cellulose production in *E. coli* [116]. Another example is the DinJ antidote which inhibits transcription of the *cspE* gene, coding for a translation enhancer of RpoS. This antitoxin directly binds to the LexA/CspE palindrome located within the *cspE* promoter [117]. The palindromic sequence that is recognized by HipB antitoxin was also identified upstream of 33 different genes, engaged in diverse processes, including persistence, metabolism, and DNA repair. Nevertheless, direct inhibition of the expression of only *relA*, *eutH*, and *fadH* genes has been experimentally confirmed to date [118]. Additionally, the HigA antitoxin of *Pseudomonas aeruginosa* was shown to bind and regulate the promoter of the *mvfR* gene that is engaged in the virulence of these bacteria [114]. Finally, the PrpA antidote of the *prpTA* TA system identified on a conjugative plasmid pMBL6842 in *Pseudoalteromonas rubra* was shown to regulate the copy number of this plasmid by directly binding to the iteron sequences within its origin of replication, thereby competing with the RepB protein for replication initiation [115].

#### 3.2.4. Examples of Cross-Induction between Different TA Systems

Another level of cross-talks within TA modules comprises cross-inductions between different TA systems in which some endoribonuclease toxins were shown to exhibit the ability to activate other TA operons via a protease-dependent or independent manner. For example, in *E. coli*, the *relBEF* operon was shown to be activated by the ectopic expression of MazF, MqsR, HicA, or HipA toxins through a protease-independent mechanism [112], while the VapCs derived from the Salmonella chromosome and Shigella plasmid stimulated YoeB toxin expression in a way dependent on the Lon protease [111].

There are also known examples where a single toxin overexpression leads to the activation of multiple toxins. The deep sea *Streptomyces* sp. SCSIO 02999 VapC toxin cross-activated several TA operons when overexpressed in *E. coli* cells, probably in an unspecific manner but partly via a Lon-dependent mechanism [109]. Additionally, ectopic overexpression of several chromosomal homologs of the VapC toxin of *M. tuberculosis* resulted in a significant increase in mRNA levels of VapCs, as well as HigB or MazF toxins [110]. In general, such phenomenon may be explained in two ways, as proposed by Kasari and colleagues [112]. Firstly, overexpression of toxins reduces protein synthesis, which in turn results in antitoxins’ protease-dependent degradation and leads to derepression and induction of the TA operons. Another possibility presumes preferential degradation of the antitoxins’ mRNA by toxins acting as RNases. This would result in the accumulation of the toxins’ mRNA that can then be translated into active proteins [110,112]. As most type II toxins are endoribonucleases, induction of these toxins impacts gene expression in general, including the expression of different TA systems, thus adding to the complexity of cellular metabolism networks.

Moreover, there is also an example of cross-regulation of expression between two different types of TA systems. The MqsR toxin of the *mqsRA* type II TA is sequence-specific RNase. It was shown that the activity of MqsR enriches mRNA of the GhoT toxin belonging to the *ghoTS* type V TA system since the latter does not possess the GCU sequence recognized by MqsR [124].

It has to be stressed that there are some shortcomings in the current studies of the interactions between different TA components. For example, they have not always been studied in their natural hosts, and sometimes the expression of toxins and antitoxins was artificially induced from different inducible promoters; some chromosomal TA cassettes were investigated when subcloned and induced on plasmids; and finally, some tested TA pairs are never observed together in their natural hosts. Thus, in several examples, the experimental set up did not reflect the natural environment and protein concentration for the tested modules. Generally, the absence of cross-talk seems to be favored by nature because even a slight imbalance in the toxin and antitoxin ratio can have catastrophic consequences for the bacterial cells. However, the presence of some confirmed cross-talks of TA systems indicates that they can give some advantages, e.g., they protect cells from undesired toxin induction. Nevertheless, in light of the potential use of TA modules in different medical or biotechnological strategies, such TAs will be used outside their natural context, will be artificially introduced, and induced in the new hosts.

## 4. Toxin-Antitoxin Systems—Practical Application in Medicine

Despite controversies on the TA systems’ biological role and uncertainty about particular mechanisms of these modules’ activation and their evolutionary pathways, attempts at the development of promising TA-based technologies for medical use have been made. Here, we divided the most propitious TA-based approaches into three categories: antimicrobial strategies (I), TA-based gene therapy strategies for viral diseases (II), and cancer (III) treatment. The TA-based medical treatment strategies have been extensively reviewed recently [125,126]; thus, in the following sections, we will mostly focus on discussing the putative impact of potential TA cross-interactions on the effects of this kind of therapy.

### 4.1. The TA-Based Antibacterial Strategies

The antibacterial strategies based on TA modules are mostly dependent on the TA system’s artificial activation. This includes approaches based on the disturbance in the TA complex formation or disruption of already formed complexes (I), inhibition of the TA systems’ expression (II), mimicking the toxin activity (III), or delivering the toxin into a pathogenic bacterial cell (IV).

Among strategies based on the disturbance in the TA complex formation, one of the most prospective research was made with the *vapBC* modules from *Mycobacterium* sp. Since homologs of this type II TA system are prevalent in a huge number (up to 50) in *Mycobacteria* [127], screens for high-affinity small molecule inhibitors against VapB-VapC interaction sites were proposed. Indeed, in 2017 the toxin-mimicking peptide that inhibits VapBC_26_ complex formation in *M. tuberculosis* was designed [128]. After a few years, this peptide was optimized by hydrocarbon α-helix stapling, leading to the synthesis of the V26-SP8 peptide that binds VapB_26_ antitoxin and thus indirectly activates VapC_26_. The performed optimization enabled for V26-SP-8 compound’s higher cell permeability; therefore, it has higher activity [129]. A similar approach was used in the case of VapBC_30_ from a model organism, *M. smegmatis.* Interestingly, it was demonstrated that the resulting V30-SP-8 peptide’s antimicrobial activity was more efficient than the activity of vancomycin [130]. Likewise, because of the VapC toxin’s strong potential for translation inhibition in pathogenic bacteria, screening for small molecules that will disrupt *M. tuberculosis* VapBC_2_ and VapBC_21_; *Klebsiella pneumoniae* VapBC complex formation was suggested [131,132].

A comparable antibacterial approach was proposed based on artificial activation of the ParE toxin with ParELC3 peptide in *E. coli* and *S. aureus* cells. The ParELC3 peptide was shown to disturb ParDE (Kis-Kid) complex formation, enabling ParE toxin to inhibit DNA gyrase activity. In this approach, the peptide was loaded into the rhamnolipid-based liposomes, which were then used as nanocarrier systems; this increased the peptide’s capability to cross bacterial membranes [133]. Still, usage of the stapled peptides or rhamnolipid-based liposomes only partially solves the problem with cell membrane permeability for toxin-activating compounds. Other issues might be related to the possibility of antimicrobial peptide degradation by the host immune response, even before reaching its target (see [134]).

Nevertheless, the antibacterial strategies described above are the response to the urgent need for developing novel types of antibiotics arising from progressing bacterial antibiotic resistance. Furthermore, it was suggested that antibiotic strategies targeting TA modules could be more selective for killing pathogenic strains than conventional antibiotics, minimizing the widespread killing of all beneficial resident flora [131]. It was also suggested that since TA modules are absent in eukaryotic cells, the risk of side effects of their activation for the host organism will be minimized [129].

Although TA-based antimicrobial technologies present undeniable advantages, they should be extensively studied in a broad context to ensure the safety of their use. For example, in contrast to VapC_2_ and VapC_21_, that block translation in bacterial cells by inactivation of the initiator tRNA^f-Met^, the VapC_26_ toxin inactivates ribosomes through cleavage at the extremely conserved sarcin-ricin loop (SLR) present in the 23S rRNA in Prokaryotes [128]. Moreover, the SLR domain is conserved in the large subunit of eukaryotic ribosomes as well. This domain is crucial for translation elongation in both Prokaryotes and Eukaryotes and may be targeted by different plant, fungal and bacterial toxins, such as ricin, α-sarcin, or Shiga toxin [135,136,137]. Thus, activation of the SLR-targeting VapC toxin could be potentially deadly for both bacterial and mammalian cells. Especially since it was already shown that some ectopically expressed toxins affect cellular processes in Eukaryotes (reviewed in [138]), including the VapC toxins of *Rickettsia* sp., which was shown to be toxic in bacteria, yeast, and mammalian cells [139]. Therefore, using *vapBC_2_* or *vapBC_21_* as an antibacterial strategy target seems to be a relatively safer option [131].

Nevertheless, it should be taken with caution that activation of a particular TA module may affect the activity of other TA systems residing within pathogenic bacteria. For example, in *M. tuberculosis*, the transcriptional cross-regulation between *vapBC_3, 11, 13, 15, 20, 27, 33_* systems and *vapBC_11, 13, 15, 20, 22, 40_* or *mazEF_6_* modules was observed [110,140]. Thereby, the possibility of cross-interactions between the targeted TA modules and the TA systems that are putatively detrimental to the host cell should be extensively studied to ensure the safety of the TA activation-based antibacterial approaches.

The problem with an unintended toxin activity towards eukaryotic cells was partially solved in an approach based on inhibition of the MazE and HipB antitoxin’s expression. The safety of this TA-based antibacterial approach was confirmed by a cytotoxicity test made with human embryonic cell line 293. The proposed strategy was based on the usage of sequence-specific antisense peptide-nucleic acid oligomers (PNA) as antitoxin-encoding gene silencers. The tested PNAs hybridized with the bicistronic TA mRNA in the antitoxin-encoding region, leading to inhibition of the antitoxin translation (probably via blocking the ribosome assembly on the mRNA) and consequently leading to mRNA degradation [141]. Although degradation of bicistronic mRNA probably results in both the antitoxin and toxin expression inhibition, it seems to be sufficient for toxin activation (details of this type of TA system’s activation mechanism are described in Section 2.2 of this review).

Interestingly, the PNAs’ inhibitory effects on bacterial growth differed depending on the target gene and the *E. coli* strain used. This could result from differences in the PNAs’ cell permeability between strains or different genetic backgrounds (e.g., possible cross-talk between activated toxins and other TA systems encoded by the targeted bacteria). Moreover, since TA modules often can be deleted with no detectable advantages or disadvantages for the bacterial cell [67], it cannot be excluded that the antitoxin targeting PNAs can provoke selective pressure driving a quick toxin encoding gene degradation. In addition, this kind of artificially induced evolutionary pressure may also lead to a relaxed specificity of non-cognate antitoxins encoded within the targeted bacterial cells. In both cases, the result would be a rapid bacterial resistance to TA-targeting PNAs. Therefore, mimicking the toxin activity seems to be a more secure antibacterial strategy.

A highly efficient antimicrobial strategy based on direct toxin-encoding gene delivery was proposed for the selective killing of antibiotic-resistant pathogenic *Vibrio cholerae* O139 strain (ABRV) [142]. In this approach, a pFW plasmid encoding two fragments of the CcdB toxin from *V. fisheri* fused with the intein-encoding fragment was designed. The use of inteins enables strict control of toxin production and avoids toxicity due to basal expression, which is especially important for the effective delivery of plasmids via conjugation between non-pathogenic *E. coli* β3914 donor and *Vibrio* sp. recipient strain. Recognition of protein fusions takes place by the intein module that carries out the splicing process, which leads to the full CcdB toxin reconstitution. Active CcdB toxin targets the DNA gyrase and blocks its activity, and in consequence, this leads to DNA replication arrest and disturbances in transcription.

In the system described above, the genes encoding hybrid proteins were placed under the control of a modified *ompU* promoter activated by the ToxR protein, which is also considered to be an indicator of *V. cholerae* pathogenicity [143,144]. Moreover, to protect the donor cells and other non-pathogenic bacteria from the toxic CcdB activity, the *ccdA* gene was introduced into the designed plasmid under the control of a modified *P_L_* promoter. The *P_L_* promoter is inhibited by the SetR protein characteristic for the *Vibrio* sp. strains harboring the SXT conjugative element, which is responsible for these bacteria’s multidrug resistance [145]. Therefore, in the mixed bacterial cultures composed of non-pathogenic *V. cholerae* O1 or *E. coli* DH5α and pathogenic ABRV O139 strains, the active, free molecules of CcdB toxin were present only in the SXT-harboring *V. cholerae* O139 cells. The high efficiency of ABRV selective killing with this elegant approach was shown using both mixed in vitro bacterial cultures and animal models characteristic for the natural environment of *Vibrio* sp.—zebrafish larvae and *Artemia salina* nauplii.

The obtained results are highly promising in the context of antibiotic-resistant *V. cholerae* strains’ treatment. However, some problems can arise from the relatively low rate of pFW plasmid conjugation efficiency under complex natural conditions [142]. On the other hand, it should be taken into account that homologs of the *ccdAB* system used here are highly prevalent within diverse bacterial species, including those that are frequently found in the aquatic environment and the digestive system of mammals (such as *E. coli* inhabiting mammals intestine or Pectobacteriaceae found in the water flowing from farmlands). It is possible that bacterial species cohabiting water or intestinal environment with pathogenic *Vibrio* strains could thus serve as the source of genes encoding antitoxins that could neutralize artificially delivered toxins in the ABRV strains, leading to rapid bacterial CcdB resistance.

Although the majority of TA-based antimicrobial strategies involve direct usage of the TA system’s components, some interesting examples of indirect TA systems’ application are also found. One of them is related to the antimicrobial activity of biogenic silver nanoparticles (AgNPs) used against *Salmonella enterica* serovar Typhimurium [146]. The proposed molecular mechanism of the AgNPs involves alterations in the reactive oxygen species production due to the interaction of AgNPs with various cellular metabolic proteins. It was suggested that in the case of the tested bacterial strain, the AgNPs bacteriostatic effect was enhanced due to these particles’ association with the *tomB-hha* system proteins. The *tomB-hha* is claimed to be a chromosomally encoded type II TA system widespread in Eneterobacteriaceae [147]. The Hha toxin interacts with HN-S proteins in *E. coli*, affecting DNA supercoiling and production of multiple proteins, thus causing a pleiotropic effect [148,149,150]. Interestingly, the toxicity of Hha in *S. typhimurium* is conditional—this toxin caused cell death only under acidic stress conditions [147]. The hydrophobic interactions between *tomB-hha* proteins and AgNPs probably affect the functional expression of this system’s components, leading to intensified bacterial cell growth inhibition via Hha toxin activity.

Similarly, it was shown recently that transcription of several type II systems in *Acinetobacter baumanii* is significantly affected by the zinc-oxide nanoparticles [151], while the nanoalumina affect *hipBA* expression in *E. coli* [152]. To conclude, although it seems possible that the TA systems could play a significant role in nanoparticle clinical application, their exact role requires further research. Specifically, this is because the TA modules’ occurrence within bacterial genomes is highly diverse, even within a single bacterial species, and does not seem to be highly conserved [7]. Therefore, the TA systems’ usage in antimicrobial strategies cannot be considered universal for all strains of pathogenic bacterial species.

### 4.2. The TA-Based Antiviral Strategies

The TA-based strategies were proposed primarily for the treatment of viral diseases that are beyond vaccination control, such as hepatitis C or acquired immunodeficiency syndrome (AIDS).

To our knowledge, only one TA-dependent approach for hepatitis C treatment has been suggested so far. That strategy was based on the usage of an antiviral agent inactivated by its fusion partner, which was linked via a viral protease cleavage site. The hepatitis C virus (HCV) belongs to the *Flaviviridaea* family, comprised of enveloped positive single-stranded RNA viruses [153]. Thus, using an RNase, such as MazF from *E. coli*, was one of the best choices for the control of its replication within the host cell. The zymotoxin, consisting of the MazF toxin and a fragment of MazE, neutralizing its activity, was designed. The TA pair was linked by an NS3 serine protease cleavage site. The NS3 protease is necessary for HCV replication, and thus, employing it to activate the zymotoxin ensures that the toxic effect of MazF will manifest only in the HCV-infected cells, leading to specific eradication of HCV-infected hepatocytes. The zymotoxin-encoding DNA was delivered into the host cells using adenovirus as a vector [154].

This elegant approach can be potentially used as a universal strategy for the treatment of other viral or bacterial infections—depending on the used TA pair and the linker type. In fact, a similar procedure was proposed for the Coxsackie virus B3 (CVB3) treatment. The CVB3 virus causes subclinical infections that can induce severe arrythmia, chronic myocarditis, and cardiomyopathy. The virus ssRNA genome encodes polyproteins which are cleaved into individual functional proteins by the 2a and 3C proteases. Using MazE-MazF fusion protein joined by the proteases cleavage sites enabled specific ablation of the CVB3-infected HeLa cells [155].

Furthermore, the bacterial *mazEF* system was used to develop a strategy for the human immunodeficiency virus-1 (HIV-1) treatment, mainly directed against latently infected CD4^+^ T cells that escape antiretroviral therapy. In this approach, a self-inactivating retroviral vector, in which the *mazF* gene was inserted under the control of the HIV-1 long terminal repeat (LRT), was constructed and used for the CD4^+^ T cell transduction. Binding of the HIV-1 Tat trans-activator protein upstream of *mazF* induced expression of the toxin, leading to viral RNA degeneration and, consequently, reduced HIV-1 replication, irrespective of the virus strain [156,157,158].

The TA-based antiviral strategies described above can be considered as a variant of gene therapy, and as such, they possess all advantages and disadvantages characteristic of this type of medical treatment. For example, a delivered transgene can potentially negatively influence normal cellular processes by competing with the natural endogenous proteins for interactions with activators, regulators, or substrates [154]. This might also be applied to the toxins and antitoxins used, which in addition, have the potential to bind DNA and thus could possibly affect the host gene expression.

There are several examples of bacterial proteins that mimic regulatory proteins present in eukaryotic cells and modulate the host cellular processes (e.g., by binding chromatin and modifying histones) (reviewed in [159]). Curiously, the phenomenon of mimicking eukaryotic proteins is more frequently observed among bacteria living in complex interactions, such as biofilms, or in bacteria exposed to protozoa attacks [159]. Interestingly, some TA systems were shown to be engaged in the processes connected with biofilm formation (reviewed in [160]).

Moreover, several proteinaceous components of bacterial TA systems share structural similarities with the domains of crucial eukaryotic proteins. For example, the KacT toxin from *K. pneumoniae*, AtaT from *E. coli*, and TacT from *S. enterica* possess GCN5-related N-acetyltransferase (GNAT) domain that can be found in the eukaryotic histone transacetylase [161]. Similarly, a large group of bacterial VapC-like RNase toxins shares the PIN-like domain that in eukaryotic cells is responsible for mRNA turnover, telomere maintenance, rRNA maturation, and tRNA processing [162,163]. Likewise, it was suggested that the mechanism of activation of the Toll/interleukin-1 receptor (TIR) domain present within the mammalian NAD^+^ hydrolase, SARM1 (responsible for the pathological axon degradation), is strikingly similar to the one observed in bacterial TA systems [164]. Furthermore, there are examples of toxins that were shown to be capable of inducing apoptosis in eukaryotic cells, such as MazF and Kid [165,166]. Therefore, despite the fact that TA modules are mainly restricted to Bacteria and Archaea, any putative cross-interactions between genetically engineered TA systems and eukaryotic host proteins should be considered in the course of the development of TA-based medical strategies.

### 4.3. The TA-Based Anticancer Strategies

The detrimental activity of several bacterial toxins was confirmed both in the procaryotic and eukaryotic cells, usually leading to apoptosis of the second ones or activation of another molecular pathway resulting in cell death [138]. As mentioned before, this attribute may be considered hazardous in the context of TA-based gene therapy. On the other hand, the cytotoxic activity of bacterial toxins delivered into mammal cells, along with differences between malignant and healthy cells in gene expression pattern, metabolic features, or micro-RNA (miRNA) expression, were exploited for the development of innovative TA-dependent anticancer approaches.

One of the TA modules that is used most extensively for this purpose is *mazEF* from *E. coli*. The MazF endoribonuclease specifically cleaves the ACA sequences within mRNA [167]. It was demonstrated that the effect of its activity could lead to tumor regression in transgenic mice, demonstrating the high potential of the *mazEF* system in modern oncotherapy [168]. Additionally, an elegant approach dependent on the MazF activity was recently proposed for the selective eradication of colorectal cancer HCT116 cells and lung cancer A549 cells [169,170]. Using one of the most frequently present in malignant cells genetic alterations (inactivation of p53 proto-oncogene tumor suppressor and RAS oncogene activation), the adenoviral vectors for *mazF* and *mazE* overexpression were designed. The *mazF* gene was located under the control of the RAS-induced modified SV40 promoter, while MazE antitoxin gene was transcribed from the modified SV40 promoter activated by the wild-type p53 gene product. It was demonstrated that in the cancer cells harboring both the p53 and RAS mutation, the amount of free MazF toxin is sufficient to induce apoptosis in over 50% of infected cells in vitro. Results of the parallel experiment performed with the wild-type cells showed over 90% cell survival rate since, in this type of cells, a proper balance between MazF and MazE was maintained, protecting cells from the MazF toxic effect. Furthermore, a 65% tumor growth inhibition of HCT116 tumors in mice was observed [170].

Although the anti-cancer strategy described above seems highly promising due to its selectivity towards malignant cells and the utility of MazF toxicity, the adenoviral vector delivery system poses several disadvantages [171]. Therefore, other types of transgene delivery systems are seriously sought. An interesting example of a *mazEF*-based anticancer strategy using bacteria-mediated delivery was proposed for viability reduction in the human breast cancer MCF7 cells, colon cancer HT29, and gastric cancer AGS cells [172,173]. In this approach, a listerial bi-vector expression and delivery system to transfer ACA-less *mazF* mRNA into the tested cell lines were used (this type of delivery system is extensively described in [174,175,176]). Depletion of the ACA sequences within the toxin-encoding mRNA ensures a relatively stable intracellular MazF level. Obtained preliminary results suggested that in vitro transfection of cell lines with ACA-less *mazF* mRNA leads to apoptosis induction, which can be used in further research focusing on developing a universal anticancer therapy based on the MazF toxin activity [172,173].

The second extensively studied TA module that was used in research focused on anticancer therapy is the type II *kis-kid* (*parDE*) system found in the R1 conjugative plasmid from *E. coli*. The Kid toxin acts as a sequence-specific ribonuclease and bacterial DNA gyrase inhibitor, leading to the SOS response induction, cell cycle arrest, and growth inhibition in bacteria [177]. This toxin was also shown to inhibit cell proliferation in a wide range of eukaryotic cells, including yeast, embryonic cells of *Xenopus laevis* amphibia [166], *Mythimna separata* insect cells [178], rabbit reticulocytes [179], *Spodoptera frugiperda* insect cells (Sf9) [178], and human HeLa and SW480 cells [166]. In the *kis-kid*-based anticancer strategy, again, the differences in gene expression between healthy and malignant cells were employed. Human cells infected with the high-risk human papilloma viruses that are the etiological cause of cervical cancer express the oncoprotein E6. This feature was used to construct a synthetic *kis-kid* system in which the Kis antitoxin intracellular level drops during E6 protein biosynthesis, leading to Kid activation [180]. There, the *kis* gene was linked to a sequence complementary to a target site for an oncogenic human miRNA. This enabled for selective killing of cancer cells overexpressing this type of miRNA [132].

Similarly, alterations in the miR-21 miRNA expression within the MCF-7 breast cancer cells were used for the development of a *yefM-yoeB-*based (type II TA system) strategy for the selective killing of malignant cells in vitro. The 3′-untranslated region of *yefM* antitoxin gene was enriched in the miR-21 target sites, which ensured the antitoxin mRNA degradation within the cancer cells, and led to selective in vitro ablation of MCF-7 cancer cells [181,182].

Besides the putative negative effects of the potential TA-cross interaction in the TA-based gene therapy described in the previous section, the proposed anticancer approaches seem to be rather safe. However, the high plasticity of the TA systems’ components should be considered in the course of the development of such medical strategies since it may lead to rapid toxin inactivation.

## 5. Practical Applications of Toxin-Antitoxin Systems in Biotechnology

Several different TA-based strategies have also been developed for diverse biotechnological applications. Here, we divided the most interesting TA-based approaches into six purpose-based categories: vector stabilization (I), counterselection (II), single protein production (III), positive selection of inhibitors (IV), containment control (V), and bacterial infections control in plants (VI). The TA-based biotechnological strategies were extensively reviewed recently [138,183,184,185], and thus in the following sections, we will mostly focus on discussing putative risks associated with their use and the impact of potential cross-interactions on possible applications.

### 5.1. Toxin-Antitoxin Modules as the Vector Stabilization Loci

Plasmids are important tools in diverse biotechnological applications. However, segregational instability of these mobile genetic elements, especially for the low copy number plasmids, may lead to their quick loss from the bacterial population. Typically, due to the metabolic burden, plasmid-harboring cells exhibit a reduced growth rate relative to the plasmid-free population. Therefore, when plasmid instability occurs, the faster-growing cells devoid of plasmid outcompete their plasmid-bearing counterparts. Thus, stable maintenance of the engineered DNA molecules comprises a significant concern in the industrial employment of microorganisms for protein or DNA production, as well as in the preparation of gene therapy constructs. For this, antibiotic-resistance genes are routinely employed; however, the use of antibiotics for medical applications and on the industry scale raises safety concerns of the world-wide regulatory authorities.

One of the tested alternatives for maintaining plasmid stability without the use of antibiotic resistance genes is toxin-antitoxin systems loci which have been successfully used as plasmid stabilization cassettes in several various approaches [186,187]. Basically, all of them rely on different stability of the toxin and the antidote components. However, the success of PSK-mediated (post segregational killing) plasmid stability depends on a sufficiently high concentration of the active toxin and on the mechanism by which the toxin affects cell survival and proliferation. Each subsequent cell division leads to the dilution of the toxin, further decreasing its ability to exert PSK. When the cell finally escapes from PSK-mediated killing, there is no mechanism to stop such plasmid-free cells from taking over the whole bacterial culture. Thus, not every TA system will be suitable as a stability determinant, and therefore the right choice is crucial here.

Several different TA modules were tested, although, in more recent applications, the *E. coli* type I *hok-sok* and enterococcal type II *axe-txe* are the ones that are frequently used. For instance, the *hok-sok* was employed to stabilize a plasmid introduced into probiotic *E. coli* Nissle 1917 to detect cancer cells in the urine, whereas the *axe-txe* was successfully used in the same bacterial strain to maintain plasmids constructed for cancer immunotherapy [188,189]. Interestingly, a study that included a mathematical model describing PSK, supported by both in vivo and in vitro experimental data, has shown that the *axe-txe* system provides greater plasmid stability than the *hok-sok* TA module [190].

For biotechnological applications, it is important to take into consideration that diverse plasmid-borne TA systems differ in their plasmid maintenance ability and specificity. Some of them are strictly specific to certain species or even strains. For instance, the *pemIK* modules were able to maintain plasmids in various strains of both *E. coli* and *K. pneumoniae*, whereas the *ccdAB* cassettes demonstrated higher specificity towards their own host strain [191]. Moreover, some TA toxins cause the host death upon loss of the plasmid, whereas some only hinder bacterial culture growth, acting as bacteriostatic agents, as was shown for the Hok and VapC toxins, respectively [192,193]. However, this effect probably mainly depends on the toxin concentration within the cell and on the timing of toxin induction under experimental conditions. Furthermore, depending on the mechanism of the TA system activation, the possibility of its application can be limited to a particular bacterial species or even a bacterial strain. For example, the lack of specific proteases engaged in particular antitoxin degradation and disruption of the TA complexes may prevent the PSK effect of the toxin. On top of that, as mentioned above, some toxins exhibit toxicity only in particular strains. For instance, the chromosomal YoeB toxin from *Agrobacterium tumefaciens* was shown not to be toxic in *E. coli*, yet it inhibited the growth of its native host [194].

In light of TA modules’ cross-talk, there is a substantial danger that potential cross-interactions between pre-existing chromosomal TA modules and the one being introduced on the plasmid may abolish cellular addiction to the plasmid. For instance, *E. coli* chromosomal *yefM-yoeB* complex was shown to cross-regulate the expression of a plasmid-encoded *axe-txe* cassette, which is often chosen as a stability module, as mentioned above [113]. Of equal importance, the TA cassette introduced on a plasmid may affect bacterial gene regulation, or some chromosomally encoded proteins can influence the expression of the plasmid TA and, in consequence, its stabilizing efficiency—as exemplified in Section 3.

### 5.2. Counterselection Systems Based on Toxins of TA Modules

The major challenge in different DNA cloning strategies is the low efficiency of inserting introduction into plasmid vectors. To increase the chances of isolating recombinant DNA molecules, two different strategies that employ toxin-antitoxin cassettes have been developed. In the cloning systems that are commercially available, StabyCloning^TM^ from Delphi Genetics Inc. and Gateway^TM^ recombination cloning system provided by Invitrogen, the CcdB toxin that targets *E. coli* DNA gyrase has been used for positive counterselection of recombinants [195]. In the first method, the toxin gene is present on the bacterial host chromosome, while the fragment of the cognate antitoxin is located within the vector. The missing part of the antitoxin gene needs to be incorporated by PCR into the insert. In this way, only recombinant DNA molecules in the right orientation of the insert will provide the active CcdA antitoxin to counteract the genome-encoded CcdB toxin [196,197]. On the other hand, the Gateway^TM^ strategy is based on the site-specific recombination system derived from bacteriophage λ. A specifically prepared vector contains a gene encoding the CcdB toxin flanked by *attP* sites, which should be replaced during cloning by the insert bearing corresponding *attB* sites incorporated in a PCR reaction. In this way, the host cell will survive only when the vector undergoes successful recombination [198,199,200].

Both of the above-described strategies rely on the CcdB toxin activity. However, there are examples of cross-interactions between chromosomally encoded and plasmid-encoded *ccdAB* modules that may result in the abolishment of the toxin’s activity [9,120]. Thus, this method is not universal, as it cannot be designed for bacterial strains that harbor cognate antitoxin; therefore, a thorough analysis has to precede the choice of the cloning system.

### 5.3. Toxin of TA Cassette in a Single Protein Production Technology

Another strategy that employs the toxin of the TA module is the single protein production system (SPP) developed by Suzuki and co-workers and later commercialized by Takara Bio company [201]. This technology is based on *E. coli* MazF toxin acting as a sequence-specific endoribonuclease that recognizes and cleaves ACA sites in vitro [167]. In this method, a gene of interest must be designed to be free of ACA sequences. Upon MazF induction, most cellular mRNAs are degraded, resulting in protein synthesis shut down except for the engineered gene of interest. This allows for efficient production of the desired protein. This technique is especially useful for toxic protein production, as well as for efficient and low-cost isotope labeling of proteins required for NMR studies [202].

Still, subsequent studies have shown that *E. coli* MazF specificity is more relaxed and that this nuclease cleaves single and double-stranded RNA in the NAC sequence, where N is preferentially U or A [203]. Moreover, some other members of the MazF family recognize different and sometimes longer sequences. For instance, *B. subtilis* MazF recognizes UACAU, while ChpBK is another MazF family toxin of *E. coli*—XACY, where X can be U or G, while Y is U, A, or G [204,205]. On the other hand, PemK, a homolog of MazF encoded by plasmid R100, preferentially cleaves single-stranded RNA at the A residue in the UAH sequence, where H is C, A, or U [206]. Finally, secondary RNA structures also play important roles in recognition by MazF since not all RNAs are cleaved by this toxin, even if recognition sites are present. On top of that, it was shown that ectopic expression of *mazF* may activate other TA systems, such as *relBE* [112]. It could be easily imagined that such a situation during protein production might affect SPP strategy because potentially induced other TA toxins could degrade mRNA of the protein to be overproduced. Additionally, again, this method is not universal as it has to be designed for a specific bacterial strain that does not possess cognate antitoxin and any other antidote which could abolish the MazF toxicity.

### 5.4. Positive Selection of Inhibitors Based on Toxin-Antitoxin System

The MazEF toxin-antitoxin system has been recently used for positive selection in a strategy of searching for inhibitors of SARS-CoV-2 main protease, Mpro, in yeast cells [207]. In this method, Alalam and co-authors took advantage of the fact that MazF displays toxicity not only toward bacteria but also eukaryotic cells, such as *Saccharomyces cerevisiae*. Thus, the toxin and antitoxin genes were cloned as a fusion separated by a DNA linker coding for the viral protease recognition sequence. In the presence of active protease, the peptide linker is cleaved. Then, the toxin and antitoxin proteins are separated, resulting in growth inhibition by the toxic component because of the higher stability of the released toxin. Therefore, any molecule that abolishes the activity of the coronavirus protease will prevent the toxin’s release, thereby promoting indicator cell growth. The authors claim that this system is suitable for screens of a large number of drug candidates, allowing for in vivo evaluation and quick adaptation for new variants.

Since the TA systems are virtually absent in the eukaryotic genomes, potential cross-interactions should not be expected. Moreover, MazF is a sequence-specific endoribonuclease that cleaves naked RNA, and therefore its toxicity would cause similar effects in different types of cells. The coding sequence of the whole DNA fusion containing the antitoxin gene, the sequence recognized by the protease, and the toxin gene, was placed under the control of one of the well-defined yeast promoters, GAL1 or MET3. Therefore, any unexpected interactions with other cellular components can be excluded. Thus, the use of the *mazEF* toxin-antitoxin module in this method appears to be safe in respect of potential cross-talk.

### 5.5. Toxin-Antitoxin Systems for Biological or Gene Containment Control

With the growing number of successful applications of genetically modified organisms (GMO) or microorganisms (GMM), the programmed or unintended release of engineered bacteria to the natural environment raises biosafety concerns. Thus, active containment systems are necessary for biotechnological applications where GMOs or GMM are used to reduce the possibility of their escape from the desired location. Biological containment reduces the viability of introduced organisms outside of the target habitat or after the completion of their desired task. On the other hand, gene containment control prevents the spread of genetic material outside the host. For the active containment system, two major factors are necessary: lethal function and control element [208]. In both strategies, different genetic approaches have been employed, including the use of toxin-antitoxin systems.

Two conditional suicide systems used *hok-sok* toxin-antitoxin module to kill bacteria when they accidentally escaped from a bioreactor. In one approach, the Hok toxin gene was cloned under the control of the tryptophan promoter, which is repressed by this amino acid. In the natural environment, where the level of tryptophan is low, derepression of *hok* expression takes place, which promotes cell death [209]. In another similar strategy, *hok* of *E. coli* R1 plasmid (*parB* locus) was cloned under the promoter of the alkaline phosphatase gene (*phoA*). Additionally, similarly, if cells were accidentally released from the bioreactor to phosphate-limited conditions, the *hok* expression would kill them [210]. Later on, more sophisticated so-called “kill switches” were created as synthetic biosafety systems that immediately prevent cell survival by inducing toxin gene expression via environmentally stimulated transcription factors. Stirling and colleagues used the *ccdAB* system to engineer two different inducible kill switches for controlling cell viability under certain conditions [211]. In one of them, the loss of function of the desired module induced cell death mediated by the CcdB toxin, while the second application was based on a low-temperature dependent promoter, and bacteria were killed at temperatures below 22 °C. Other examples include promoters that respond to different ions, controlling expression of some TA toxins in cyanobacterial species [212], and several type I and type II TA systems that were described and tested in kill switches in plant-beneficial *Pseudomonas fluorescens* [213].

The kill switch strategy employing TA modules was also tested in different eukaryotic species. For instance, the RelE toxin gene was successfully cloned under a glucose-repressed promoter in *S. cerevisiae* as a host. In the glucose-limited environment, the transgenic yeast were killed by this toxin, showing that such a strategy could be potentially used to control the fermentation process in the industry [214]. More recently, there were several successful attempts to express TA systems in plants. It was shown that expression of YoeB derived from *S. pneumoniae* cloned under a strictly inducible promoter was lethal to the plant model, *Arabidopsis thaliana*. The toxin induction resulted in plant development defects and necrotic symptoms, which were followed by plant death from the detrimental effects of YoeB as an endoribonuclease in both the bacterial and eukaryotic cells [215]. In a different study, the MazF toxin was expressed in a specific type of cell in the plant *Nicotiana tabacum*, called the tapetum, which are essential for the development of pollen grains. In parallel, the MazE antitoxin was expressed in the other plant tissues to protect them from the toxin’s activity. In this way, researchers were able to obtain male sterility in tobacco plants [216].

However, as underlined by the authors of the studies mentioned above, inhibition of cell growth in these systems is not complete, indicating that some cells may escape the toxin’s attack, and therefore control of the spread of transgenic organisms would not be risk-free. Moreover, most of the existing biocontainment strategies are not effective enough to achieve both low escape frequency and high efficiency. Mutations inactivating the toxin usually occur within the toxin gene or in the promoter controlling its expression. Therefore, for the long-lasting effect which is necessary for some of these applications, such a situation raises serious concerns. The escape effect is not surprising, considering the increased fitness of mutant strains harboring inactivated toxin genes [213]. Additionally, as mentioned in one of the previous sections, there are toxins that were shown to be lethal only for particular bacterial species [194]. This is of exceptional concern for a containment system targeted toward random hosts.

### 5.6. Toxin-Antitoxin Used to Control Bacterial Infections of Plants

Recently, the MqsR toxin of *mqsRA E. coli* TA cassette was used in a strategy to fight bacterial infections of citrus plants. The toxin’s gene was introduced into the genomes of different citrus plants via the *Arabidopsis tumefaciens* transformation strategy. In the construct, MqsR was fused to a signal peptide to direct the toxin outside of the plant cell. Next, these transgenic plants were infected by bacterial pathogens, *Xanthomonas citri*, and *Xylella fastidiosa*, causing citrus canker and citrus variegated chlorosis, respectively. Plants overexpressing the MqsR toxin were shown to have reduced symptoms of the disease, indicating pathogen cell death mediated by this toxic protein [217]. This finding opens new and promising possibilities to prevent bacterial crop infections that could be of great economic importance.

## 6. Concluding Remarks

Although cross-interactions between bacterial toxin-antitoxin systems do not seem to be the main factor affecting the TA systems’ biology, they are one of the crucial elements that drive TA module evolution. In the natural environment, the TA systems’ cross-talk usually leads to such systems differentiation. However, in large part, of the proposed biotechnological and medical TA-based applications, an opposite trend occurs. The main reason for this is using components of the TA systems that naturally do not coexist within a single cell, which raises the probability of their cross-talk and forces selective pressure, which is opposite to the one occurring in the natural environment (e.g., by artificial activation of the toxin). Since unintentional TA cross-interactions can significantly affect the outcome of the TA-based methods, these kinds of side effects should be carefully examined before the implementation of TA-based approaches in medicine and biotechnology.

## Figures and Tables

**Table 2:** Examples of cross-talk between proteins belonging to different homologous and non-homologous type II toxin-antitoxin systems, as well as TA cross-regulations with other cellular genes.

Original TA Location	Type of Cross-Talk	Examples of Cross-Talk
chromosome/chromosome	homologous TAs cross-interactions	*M. tuberculosis* MazEF and RelBE [100,101,102] *B. longum* MazEF [103] *Y. pestis* RelBE [104]
chromosome/chromosome	non-homologous TAs cross-interactions	*B. longum* MazEF-RelBE [103], *M. tuberculosis* MazEF-VapBC [100]
chromosome/plasmid	homologous TAs cross-interactions	*E. chrysanthemi*—P1 CcdAB, *E. coli* O157:H7—pO157 CcdAB *E. coli* O157: H7—pB171-like AtaRT [80] *E. coli* MazEF—R1 Kis/Kid [105]
plasmid/plasmid	non- homologous TAs cross-interactions	*E. coli* R1 Kis/Kid—F1 CcdAB [106]
chromosome/phage	homologous TAs cross-interactions	*V. cholerae* Phd/Doc—P1 Phd/Doc [107]
chromosome/phage	non- homologous TAs cross-interactions	*E. coli* RnlAB—T4 Dmd [108]
plasmid/phage	non- homologous TAs cross-interactions	*E. coli* O157:H7 pOSAK1 IsoAB—T4 Dmd [88]
chromosome or plasmid or phage	TAs-indirect cross-activation	*Streptomyces* and *M. tuberculosis* VapBC-Lon-several TAS [109,110] *Salmonella* and *Shigella* VapBC—Lon-YefM/YoeB [111] *E. coli* RelBEF—Lon independent-several TAS [112]
chromosome or plasmid or phage	TAs-direct cross-regulation	*S. oneidensis* ParE-CopA-promoter of MR-1 PemIK [76] *E. faecium* pRUM Axe/Txe-promoter of *E. coli* YefM/YoeB and vice versa [113]
chromosome or plasmid or phage	TA-direct regulation of unrelated protein	*P. aeruginosa* HigA-*mvfR* promoter [114] *P. rubra* PrpA-*ori* iterons [115] *E. coli* MqsA-*csgD* promoter [116] *E. coli* DinJ-*cspE* promoter [117] *E. coli* HipB-*relA*, *eutH* and *fadH* promoters [118]

## Data Availability

Not applicable.
